# Structure basis for allosteric regulation of lymphocytic choriomeningitis virus polymerase function by Z matrix protein

**DOI:** 10.1093/procel/pwad018

**Published:** 2023-04-11

**Authors:** Lu Liu, Panpan Wang, Aijun Liu, Leike Zhang, Liming Yan, Yu Guo, Gengfu Xiao, Zihe Rao, Zhiyong Lou

**Affiliations:** MOE Key Laboratory of Protein Science, School of Medicine, Tsinghua University, Beijing 100084, China; School of Life Sciences, Peking University, Beijing 100080, China; Kobilka Institute of Innovative Drug Discovery, School of Life and Health Sciences, The Chinese University of Hong Kong, Shenzhen 518100, China; State key laboratory of Virology and National Virus Resource Center, Wuhan Institute of Virology, Center for Biosafety Mega-Science, Chinese Academy of Sciences, Wuhan 430071, China; MOE Key Laboratory of Protein Science, School of Medicine, Tsinghua University, Beijing 100084, China; State Key Laboratory of Medicinal Chemical Biology, College of Life Sciences and College of Pharmacy, Nankai University, Tianjin 300350, China; State key laboratory of Virology and National Virus Resource Center, Wuhan Institute of Virology, Center for Biosafety Mega-Science, Chinese Academy of Sciences, Wuhan 430071, China; MOE Key Laboratory of Protein Science, School of Medicine, Tsinghua University, Beijing 100084, China; State Key Laboratory of Medicinal Chemical Biology, College of Life Sciences and College of Pharmacy, Nankai University, Tianjin 300350, China; National Laboratory of Biomacromolecules, CAS Center for Excellence in Biomacromolecules, Institute of Biophysics, Chinese Academy of Sciences, Beijing 100105, China; Shanghai Institute for Advanced Immunochemical Studies and School of Life Science and Technology, ShanghaiTech University, Shanghai 200120, China; Innovation Center for Pathogen Research, Guangzhou Laboratory, Guangzhou 510005, China; MOE Key Laboratory of Protein Science, School of Medicine, Tsinghua University, Beijing 100084, China

## Dear Editor,

The *Arenaviridae* family (recently assigned to the *Bunyavirales* order) is a group of emerging viruses that include causative agents of severe hemorrhagic fevers with high mortality in humans (de la Torre, 2009). Lymphocytic choriomeningitis virus (LCMV) is the prototypic member of the *Arenaviridae* family and belongs to the Old World (OW) arenavirus together with Lassa virus (LASV), which are distinct from the New World (NW) arenavirus [e.g. Machupo virus (MACV) and Junin virus (JUNV)]. LCMV infection in the fetus and newborn results in severe impairment of brain development associated with sensory loss and mental retardation and is also known to be associated with severe systemic infection with high mortality in transplantation patients (Palacios et al., 2008).

As a segmented negative-sense single-stranded RNA virus (sNSRV), arenavirus encodes a large polymerase (L) to form a ribonucleoprotein complex (RNP) together with the genomic-length RNA encapsidated by viral nucleoprotein (NP) and facilitate virus replication and transcription. Arenavirus also encodes a RING finger Z matrix protein as a regulator of RNA synthesis. Previous studies have shown that the arenavirus Z protein forms a species-specific complex with the L protein and inhibits RNA synthesis initiation by impairing the catalytic activity of the L protein, which is known to be essential for the balance of infection and further initiation of virion assembly; however, the mechanism remains unclear. Moreover, the Z protein has also been found to mediate key host–virus interactions for efficient arenavirus proliferation (Borden et al., 1998; Volpon et al., 2010).

Here, we determined the cryo-EM structures of LCMV-L and its complex with the Z protein at atomic resolution. These structures show that the binding of Z at the bottom of the interface between the core lobe of the PA-like domain and the palm subdomain of the RdRp domain partially hinders the exit of nascent RNA products and results in several distinct conformational shifts of L structural elements. These results reveal a mechanism for the allosteric regulation of arenaviral polymerase activity by Z and indicate a strategy for antiviral development.

Full-length LCMV-L and Z were individually purified and incubated at 4°C overnight to reconstruct the L–Z complex. The sample was plunge-frozen on Quantifoil R1.2/1.3 Cu grids, and a cryo-EM dataset was collected using a Titan Krios 300 kV equipped with a K2 Summit detector (Fig. S1; Table S1). After motion correction, contrast transfer function (CTF) estimation, iterative rounds of 2D classification, and heterogeneous 3D refinement, a total of 405,657 particles remained in two classes representing the individual L and the L–Z complex, respectively. Homogeneous 3D refinement of these two particle stacks resulted in a 3.4-Å map for the individual L protein and a 3.6-Å map for the L–Z complex. The models of LCMV-L and the bound Z were manually constructed under the guidance of LASV L (Peng et al., 2020) and the crystal structure of LASV-Z (Hastie et al., 2016).

**Figure 1.**
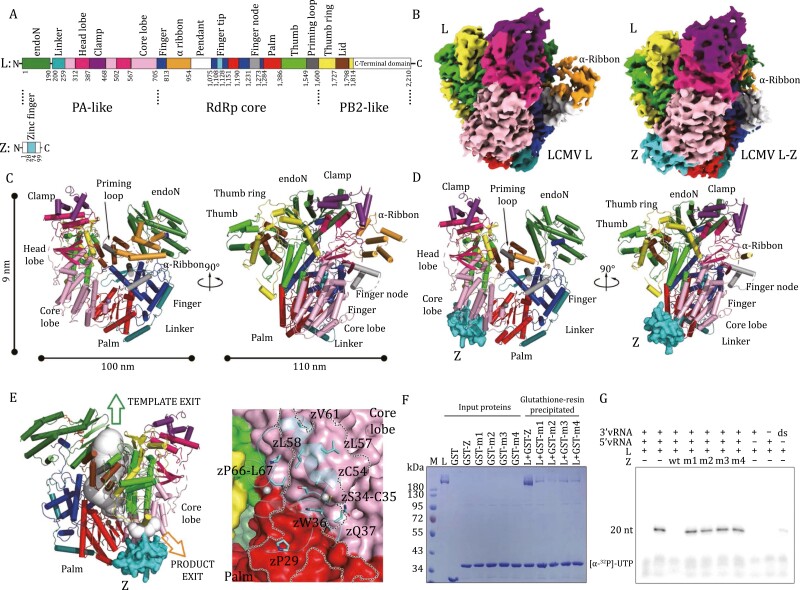
**Structures and interaction of LCMV-L and L–Z complex.** (A) Domain organization of LCMV-L and LCMV-Z. The endoN domain is shown in forest green; the linker region is shown in deep teal; the core lobe domain is in light pink; the head lobe is in hot pink; the clamp is in violet; the Finger is in blue; the α-ribbon is in bright orange; the Finger tip is in cyan; the palm is in red; the Finger node is in gray; the Thumb is in green; the priming loop is in dark gray; the Thumb ring is in yellow and the lid is in brown. The interdomain borders are labeled with residue numbers. The pendant domain with invisible density is colored white. (B) Cryo-EM densities of LCMV-L and L–Z complex. The densities for the L and L–Z complex are shown in the same orientation. Domains are colored as the same in (A). (C and D) Ribbon diagrams of LCMV-L and L–Z complex polypeptide chains are shown in two views. Domains are colored as the same in (A). A cation bound near the RdRp catalytic center is shown as a gray sphere. (E) The binding of Z blocks the product exit tunnel for the releasing nascent RNA product. L is shown in cartoon, and the domains are colored the same as in (A). The template exit tunnel is shown in forest green, and the product exit tunnel is in bright orange. The Z protein is shown as a surface model that binds at the bottom of the interface between the core lobe of the PA-like domain and the palm subdomain of RdRp, blocking the exit of the nascent RNA product. The LCMV L–Z interaction sites are shown in (A, right). (F) GST pull-down assay. LCMV-L (L), GST tagged LCMV-Z (GST-Z), free GST (GST) or GST tagged LCMV-Z mut1, LCMV-Z mut2, LCMV-Z mut3, and LCMV-Z mut4 (GST-m1, GST-m2, GST-m3, and GST-m4) were incubated in the designated combinations before being added into glutathione resin. The resin was washed three times, and the components were eluted and analyzed by 10% denaturing SDS/PAGE staining with colloidal Coomassie. (G) RNA products resulting from the *in vitro* polymerase assay. 3ʹ or 5ʹ vRNAs are conserved 3ʹ/5ʹ terminal RNAs of the viral genome S segment, and ds-RNAs were 3ʹ and 5ʹ vRNAs pre-annealed at 65°C before use.

LCMV-L displays a similar architecture to LASV and MACV-L proteins (Peng et al., 2020), with r.m.s.d of 1.882 and 2.729 for 1,282 and 1,110 aligned Cα atoms, respectively, which is consistent with their relatively high amino acid similarities (Fig. S2). According to the structures of LASV/MACV-L, the polypeptide of LCMV-L can be divided into three parts: the N-terminal PA-like region, the RdRp region (PB1-like domain), and the C-terminal PB2-like region (Figs. 1A, S2 and S3). We assigned residues to LCMV-L domains as follows (Figs. 1A, S2 and S3): residues 1–190 belong to the endonuclease (endoN) domain; a linker region spans residues 200–259; residues 260–705 belong to a PA-C-like domain; residues 706–1,600 belong to the RdRp core; residues 1,601–1,814 belong to a PB2-like domain, in which residues 1,601–1,727 and 1,799–1,814 belong to the Thumbing ring domain; and residues 1,728–1,798 are a lid domain. The RdRp region is caught in the middle by the PA-like domain, where the PA-C-like region tightly binds to the Thumb domain of the RdRp region and the endoN domain is on the other side (Fig. 1B–D). The C-terminal residues 1,815–2,210 cannot be observed in the cryo-EM densities of LCMV-L and the L–Z complex, which is similar to that observed in monomeric LASV/MACV-L and L–Z (Peng et al., 2020). This portion is supposed to adopt the cap-binding domain that may have a flexible architecture, as observed in influenza virus polymerase (Lehmann et al., 2014; Peng et al., 2020).

In the LCMV L–Z complex structure, L and Z form a heterodimer with a molar ratio of 1:1. The final model of the LCMV L–Z complex includes residues 1–1814 of L and residues 28–74 of Z (Fig. 1A). The Z protein was found to bind at the bottom of the interface between the core lobe of the PA-like domain and the palm subdomain of the RdRp core (Fig. 1E). The position of Z partially blocks the product exit tunnel for the releasing nascent RNA product (Fig. 1). Although the full-length Z was used to generate the L–Z complex, only its zinc finger domain can be built in the complex structure, suggesting that its N-/C-terminal portions are flexible and do not participate in the interaction with L. The zinc finger domain of LCMV-Z contains a single α-helix and two β-strands and harbors two zinc ions by residues C32–C35–C51–C54 and C45–H48–C65–C68 to form two zinc fingers, which are conserved with that observed in LASV-Z (Hastie et al., 2016) (Fig. S4).

The LCMV L–Z interaction comprises a set of hydrophobic interactions, hydrogen bonds, and van de Waals interactions with a large intermolecular contact surface of ~780 Å^2^, which is 20% of the ~4,000 Å^2^ total area of the molecular face of Z (Table S2). The interacting residues of Z are located on the surface at the side of the two zinc-binding sites, including P29, S34, C35, W36, Q37, C54, L57, L58, V61, P66 and L67 (Figs. 1E and S2). A previous study showed that LASV-Z interacts with eIF4E through Z residues F30, K32, S33, W35, N38, and K39 (Volpon et al., 2010), where the area overlaps with the LCMV L–Z interface, indicating that the interaction of Z with L and eIF4E is incompatible (Fig. S6A). Moreover, the crystallographic structure of LASV-Z and biochemical analysis of LCMV-Z presented oligomerized states of their Z, and the suggested oligomerization intermonomer interface also largely coincided with the L–Z interface (Hastie et al., 2016), indicating that a transition of Z from oligomer to monomer might be a prerequisite for its binding with L (Fig. S6B).

To validate the impact of the interacting residues on the L–Z interaction, we substituted P29, S34, C35, W36, and Q37 with alanine residues in mutant-1; L57, L58, and V61 with alanine residues in mutant-2; P66 and L67 with alanine residues in mutant-3; and the mutations in both mutant-1 and mutant-2 in mutant-4 and checked the interactions between L and the wild-type (wt) or the mutated Z proteins in the GST pull-down assay. The results showed that all four mutants significantly attenuated the binding of LCMV-Z to L (Fig. 1F). Earlier work on MACV-L indicated that 5ʹ RNAs are bound as single-stranded ligands rather than a duplex structure with 3ʹ RNAs, and the integrated panhandle structure (dsRNA) influences the capability of RNA synthesis of L (Kranzusch et al., 2010). We first characterized the impact of different RNA substrates on LCMV-L enzymatic activity *in vitro* (Fig. 1G). We used 19-nt and 20-nt RNAs corresponding to the conserved 3ʹ/5ʹ terminal RNA of the viral genome S segment as templates (Fig. S7B). The results showed that LCMV-L activity was weak when using the individual 19-nt 3ʹ vRNA or 20-nt 5ʹ vRNA as the template (Fig. 1G). In contrast, when we provided both the 20-nt 5ʹ-vRNA and 19-nt 3ʹ-vRNA in the enzymatic reaction assay, the activity of LCMV-L was significantly enhanced (Fig. 1G). Moreover, if the 3ʹ-vRNA and 5ʹ-vRNA were annealed to form a hybrid and used as a double-stranded promoter RNA in the LCMV-L activity assay, the activity of LCMV-L was much weaker than that provided by the individual 3ʹ-vRNA and 5ʹ-vRNA in the activity assay, although this activity was slightly stronger than that used 3ʹ-vRNA or 5ʹ-vRNA alone in the assay. These results are consistent with previous studies of MACV-L (Kranzusch et al., 2010; Pyle and Whelan, 2019), suggesting that the impact of RNA primers on arenaviral L activity is conserved in mammarenaviruses.

Furthermore, we checked whether the substitutions of interacting residues of LCMV-Z may affect the inhibition of polymerase activity of L. Single-stranded 19-nt 3ʹ vRNA and 20-nt 5ʹ vRNA above were used in all future *in vitro* assays. The results show that the binding of the wild-type Z protein to L significantly diminishes the catalytic activity of L, but the mutants restore the function of L (Fig. 1G).

**Figure 2.**
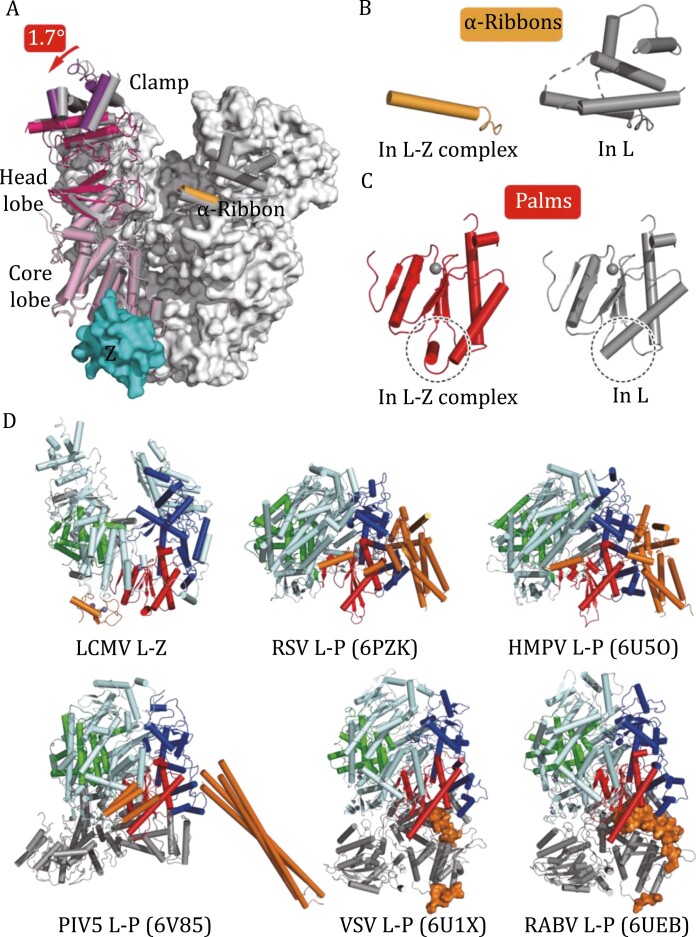
**The conformational change mediated by Z and the comparison of LCMV and nsNSRV L-accessory protein complexes.** (A) Orientation shifting after Z binding. The binding of LCMV-Z results in several conformational changes in L, the core lobe, head lobe, and clamp domain of the PA-like region have a 1.7° orientation shifting apart from the RdRp core with the Z binding site as the fixed point. (B) The sharp contrast of α-ribbon motif. In sharp contrast, with the binding of Z, only one α-helix can be observed, and most parts of the α-ribbon motif lack interpretable cryo-EM density compared with L. (C) Conformational change in palm subdomain. An additional conformational change of an α-helix in the palm subdomain can be observed in the LCMV L–Z complex. (D) Comparison of LCMV and nsNSRV L-accessory protein complexes. Structures of the LCMV L–Z complex, RSV (6PZK) and HMPV (6U5O) L–P complex, PIV5 L–P complex (6V85), VSV L–P (6U1X) and RABV L–P complex (6UEB) are aligned by their RdRp core and are shown as cartoons in the same orientation. The finger, palm, and thumb subdomains of all L proteins are colored blue, red, and green, respectively. The accessory proteins bound to L are colored orange. Note that for clear representation, the fragment of P bound to VSV L and RABV L is covered with a molecular surface. The PA-like region in LCMV-L and the PRNTase region in the other five L proteins are colored pale cyan. The PB2-like region in LCMV-L and the CD-MTase-CTD region in the other five L proteins (if present) are colored gray.

The binding of LCMV-Z results in several conformational shifts of L (Fig. 2). First, compared with apo LCMV-L, the core lobe, head lobe, and clamp domain of the PA-like region have a 1.7° orientation shifting apart from the RdRp core with the binding site of Z as the fixed point (Fig. 2A). Because Z binds at the bottom of the interface between the core lobe of the PA-like domain and the palm subdomain of the RdRp domain, we propose that the insertion of Z to the interface of the PA-like and RdRp domains leads to this orientation shifting, although this is a slight shifting. Second, the RdRp domain of LCMV-L has an integrated α-ribbon motif comprising five visible α-helices, which has been shown to play a key role in binding with and translocating template-primer hybrids to the polymerase catalytic center (Gerlach et al., 2015) in the absence of the Z protein (Fig. 2B). In sharp contrast, with the binding of Z, only one α-helix can be observed, and most parts of the α-ribbon motif lack interpretable cryo-EM density, suggesting that these parts have a flexible architecture (Fig. 2B). Finally, upon binding with Z, an additional conformational change of an α-helix in the palm subdomain can be observed in the LCMV L–Z complex (Fig. 2C).

The RING finger protein Z of arenavirus is a multifunctional protein that has been implicated in many facets of the arenavirus viral life cycle, including regulating viral RNA synthesis, interaction with host cellular factors, arrangement of viral assembly and budding, and antiviral signaling (Borden et al., 1998). Our atomic resolution complex structure of LCMV showed a direct interaction between L and Z, revealing allosteric regulation by the Z protein, and we proposed a model of this regulation (Fig. S8). The Z protein was found to bind at the bottom of the interface between the core lobe of the PA-like domain and the palm subdomain of the RdRp core. Compared with apo LCMV-L, the core lobe, head lobe, and clamp domain of the PA-like region have a 1.7° orientation shifting apart from the RdRp core with the binding site of Z as the fixed point. With the binding of Z, only one α-helix can be observed, and most parts of the α-ribbon motif lack interpretable cryo-EM density, suggesting that these parts have a flexible architecture (Figs. 2B and S8). Moreover, an additional conformational change of an α-helix in the palm subdomain can be observed in the LCMV L–Z complex (Fig. 2C). In summary, the insertion of the Z protein induces a conformational change in LCMV-L.

Virus polymerases typically require binding cofactors to achieve efficient replication and transcription. For non-segmented negative-sense single-stranded RNA viruses (nsNSRVs) (*Mononegavirales* order), mononegaviruses employ a phosphoprotein (P) to tether L to the nucleoprotein-RNA complex and act as a chaperone that prevents the association of nascent N (N^0^) with host cell RNAs. Current structural insights into NSRV-encoded L in complex with viral accessory proteins are largely acquired in nsNSRVs (Fig. 2D). Studies of the L–P complexes from vesicular stomatitis virus (VSV) and rabies virus (RABV) (in the *Rhabdovirdae* family) have revealed that the P protein attaches around the CTD, CD, and RdRp domain and locks the MTase-CD-CTD in a closed state that represents a preinitiation conformation (Liang et al., 2015; Horwitz et al., 2020). In human respiratory syncytial virus (RSV) and human metapneumovirus (HMPV) (in the *Pneumoviridae* family) L–P complexes, tetrameric P mainly binds to the finger subdomain of the core RdRp domain to elevate polymerase activity (Pan et al., 2020). In the parainfluenza virus 5 (PIV5, *Paramyxoviridae* family) L–P complex, the C-terminal X domain (XD) of P binds to the surface of L with a helix near the NTP entry tunnel (Abdella et al., 2020). In all of these structures, the binding of viral accessory protein does not result in a distinct conformation shift of L. Compared with these structures, LCMV-Z binds at a distinct position, and its binding to L results in the instability of the α-ribbon and the orientation shift of regions in the PA-like domain. Further structural study of other sNSRV L-regulator complexes is warranted to dissect the evolutionary relationship in the viral accessory protein to regulate the function of L.

Previous functional studies on MACV-L have shown that the Z protein inhibits viral RNA synthesis in a species-specific manner. The high-resolution complex structure of LCMV L–Z clearly shows the interface. A highly conserved tryptophan (W) residue located at position 36 in LCMV-Z, which is highly conserved for all mammarenaviruses (OW and NW), contributed to Z inhibitory activity (Cornu and de la Torre, 2002). In addition to W36, C35, the second conserved site of OW and NW, is also involved in the L–Z interaction. Furthermore, P29, S34, L57, L58, V61, and P66 of LCMV-Z in the L–Z interface are conserved in both LCMV-Z and LASV-Z but not conserved in MACV-Z (Fig. S2B), suggesting differences between OW and NW, which may explain the previous observation that LCMV-Z cannot inhibit RNA synthesis of MACV-L as efficiently as MACV-Z. Our results provide structural insights and together with biochemical studies demonstrated that arenavirus Z proteins may not function as broadly active inhibitors and must instead use a species–specific interaction.

Arenaviruses pose a biodefense threat, and six of them, including LASV and LCMV, are Category A agents. However, the therapeutic approach is limited to the use of ribavirin, which is only partially effective and associated with significant side effects (de la Torre, 2009). The interface of the L–Z interaction presents a deep hydrophobic groove on the surface of L, providing a potential new site for the development of anti-arenavirus reagents. The compound that binds to this site with higher binding affinity would compete for the binding of Z to L and lock L in the inactive state. Moreover, the species specificity of the L–Z interface might lead to the discovery of species-specific anti-arenavirus inhibitors.

In conclusion, the atomic structures of the LCMV-L and L–Z complex reported here present insight into the polymerase-regulator complex encoded by sNSRV and reveal an allosteric regulatory mechanism of polymerase activity by Z in the *Arenaviridae* family. These findings will further the understanding of the regulation of arenaviral replication machinery and highlight the potential to discover allosteric polymerase inhibitors against arenavirus infection.

## Supplementary Material

pwad018_suppl_Supplementary_Materials

## Footnotes

The atomic coordinates of the apo LCMV-L and LCMV L–Z complex have been deposited in the Protein Data Bank (PDB) with the accession codes 7X6S and 7X6V. The corresponding maps for apo LCMV-L and LCMV L–Z complex have been deposited in Electron Microscopy Data Bank (EMDB) with codes EMD-33026 and EMD-33028 respectively.

We thank the Computing and Cryo-EM Platforms of Tsinghua University, Branch of the National Center for Protein Sciences (Beijing) for cryo-EM sample preparation and screening and the Cryo-EM Facility Center of Southern University of Science and Technology (Shenzhen) for providing facility support. This work was supported by the National Natural Science Foundation of China (32188101 and U20A20135), the National Program on Key Research Project of China (2020YFA0707500), and Tsinghua University Spring Breeze Fund.

Lu Liu, Panpan Wang, Aijun Liu, and Leike Zhang contributed equally to this study.

Z.L., Z.R., and G.X. conceived the project and designed the experiments. L.L., P-P.W., L.Y., and Y.G. expressed and purified the proteins. L.L. and P-P.W. prepared the cryo-EM sample. L.L., P-P.W., and A.L. collected, processed the cryo-EM data, and built the model. L.L. and L.Z. performed the polymerase activity experiments. Z.L. and L.L. wrote the manuscript. All authors discussed the experiments, read, and approved the manuscript.

This article does not contain any studies with human or animal subjects performed by any of the authors. All authors declare no conflict of interest.
