# Supplementary material for: Structure basis for allosteric regulation of lymphocytic choriomeningitis virus polymerase function by Z matrix protein
Source: Protein Cell. 2023 Apr 11;14(9):703–7. doi: 10.1093/procel/pwad018 (PMC10501185; doi:10.1093/procel/pwad018)
Supplement: pwad018_suppl_Supplementary_Materials [file pwad018_suppl_supplementary_materials.pdf]

Supplemental materials

Methods

Protein production and purification

LCMV-L protein production and purification (strain Armstrong) with an N-terminal 6× His tag and a C-terminal 2× Strep tag was expressed in Sf9 insect cells using the pFASTbac HTB transfer vector (Invitrogen, USA). Sf9 cells expressing polymerase were lysed by sonication in Buffer A containing 50 mM NaH2PO4 (pH 8.0), 600 mM NaCl, 10% (v/v) glycerol, 4 mM MgCl2, and 0.2% NP-40 supplemented with phenylmethylsulfonyl fluoride (PMSF) and 1 U/ml benzonase (Millipore, USA). After clarification by high-speed centrifugation, the cell lysate was loaded onto a Strep column (GenScript, USA). After washing three times with Buffer B (100 mM Tris-HCl pH 8.0, 300 mM NaCl, and 1 mM EDTA) to remove nonspecifically bound protein, the target protein was subsequently eluted using Buffer C containing 100 mM Tris-HCl pH 8.0 and 300 mM NaCl supplemented with 1 mM EDTA and 50 mM biotin. The eluted L protein was pooled and further purified using a heparin column (GE Healthcare, USA) in 20 mM HEPES (pH 7.5) with 2 mM dithiothreitol (DTT). The L protein was eluted from the heparin column using a gradient of NaCl to a final concentration of 600 mM. The fractions near the maximum height of the peak were combined, concentrated with an Amicon Ultra Centrifugal Filter (Millipore, USA), flash-frozen and stored at -80 °C.

Full-length LCMV-Z (strain Armstrong) was constructed into pGEX-6p-1 with a GST tag followed by an HRV 3C site in the N-terminus, and LCMV Z mutants were generated using site-directed mutagenesis. The plasmids were transformed into E. coli BL21 (DE3), and the transformed cells were cultured at 37 °C in LB media containing 100 mg/L ampicillin. After the OD600 reached 0.6, the culture was cooled to 16 °C and then induced with 0.5 mM IPTG (supplemented with 100 mM ZnSO4). After overnight induction, the cells were harvested through centrifugation, and the pellets were resuspended in lysis buffer (50 mM Tris-HCl, pH 8.0, 300 mM NaCl) supplemented with phenylmethylsulfonyl fluoride (PMSF) and homogenized with an ultrahigh-pressure cell disrupter at 4 °C. The insoluble material was removed through centrifugation at 13,000 rpm. The fusion protein was first purified by GST affinity chromatography and eluted through on-column tag cleavage by Prescission protease. The eluate was further purified by a Hitrap Q ion-exchange column (GE Healthcare, USA), where pure LCMV Z was collected in the flow through. Following the ion-exchange purification, the LCMV-Z was purified onto a Superdex 200 10/300 Increase column (GE Healthcare, USA) in a buffer containing 10 mM Tris-HCl, pH 7.5, and 150 mM NaCl. The peak was analyzed by SDS–PAGE and concentrated with an Amicon Ultra centrifugal concentrator (Millipore, USA).

Cryo-EM sample preparation and data collection

To prepare L-Z protein complexes, LCMV L polymerase and Z protein were incubated

with a molar ratio of L:Z=1:1 at 4 °C for 2 h. The mixture was then loaded onto a Superose 6 10/300 GL (GE Healthcare, USA) column equilibrated with 20 mM HEPES, pH 7.5, 500 mM NaCl, and 1 mM dithiothreitol (DTT). The complex fractions were collected and concentrated to 1 mg/ml. An aliquot of 3 μl of sample at 1 mg/ml was loaded onto a glow-discharged Quantifoil R1.2/1.3 Cu grid (400 mesh) (Quantifoil, Germany). The grid was then blotted for 3.5 s with a blot force of 0 in 100% relative humidity and plunge-frozen in liquid ethane using a Vitrobot Mark IV (FEI, USA). Cryo-EM data were collected with a 300-kV Titan Krios electron microscope (FEI, USA) and a K2 Summit direct electron detector (Gatan, USA). A series of micrographs were recorded as movies (32 frames, 5.76 s) under super-resolution counting mode at a calibrated magnification of 130,000×, resulting in a pixel size of 0.54 Å per pixel. Raw image of LCMV L-Z particles in vitreous ice recorded at -1.5 to -2.5 μm defocus. Statistics for the data collection and refinement are summarized in Supplementary Table 1.

Cryo-EM image processing

Individual frames from each micrograph movie were aligned and averaged using MotionCor2 (Zheng et al., 2017) to produce drift-corrected 2× binned images. Particles were picked and selected in RELION 3.0 (Scheres, 2012) from dose-weighed micrographs with a box size of 220 pixels, and the contrast transfer function (CTF) parameters were estimated using CTFFIND4 (Rohou and Grigorieff, 2015). Subsequent steps for particle picking, 2D and 3D classification and the reconstruction of the final selected particles were performed with THUNDER and CryoSparc (Punjani et al., 2017; Hu et al., 2018). A total of 405,657 particles remained in two classes representing the individual L and the L-Z complex. Homogeneous 3D refinement of these two particle stacks resulted in a 3.4-Å map for the individual L protein and a 3.6-Å map for the L-Z complex. The final resolution was assessed using the gold-standard FSC criterion (FSC = 0.143) with RELION 3.0 (Scheres, 2012). The data quality and the particle distribution were also analyzed by 3DFSC (Tan et al., 2017) and cryo-EF (Naydenova and Russo, 2017).

Model building and refinement

To solve the structure of the LCMV-L protein, the structure of the RdRp region of LASV-L (Peng et al., 2020) and the crystal structure of LACV-Z (Hastie et al., 2016) were individually manually placed, and were rigid-body fitted into the cryo-EM density map with UCSF Chimera (Pettersen et al., 2004). Other parts of the model were manually built in Coot (Emsley et al., 2010) with the guidance of the cryo-EM density, except for several loops or the most C-terminal regions (residues 191-199, 307-314, 356-369, 401-410, 466-471, 507-523, 785-831, 848-856, 883-887, 919-934, 955-1080, 1250-1254, 1549-1566, 1579-1601, 1699-1731, 1757-1771 and 1815-2210) of LCMV-L and the N/C-terminal region (residues 1-26 and 75-90) of LCMV-Z that could not be traced due to a lack of

interpretable densities. Manual model building was subsequently performed using Coot in combination with real space refinement with Phenix (Afonine et al., 2012) to replace the corresponding amino acids in the model with those from LCMV L and LCMV L-Z. The density maps were kept constant during the entire fitting process, and only the atomic coordinates were subjected to refinement. The data validation statistics are shown in Supplementary Table 1.

GST pull-down assay

LCMV L (5 μg) was incubated with GST-tagged Z or GST alone (3 μg) in binding buffer (PBS with 250 mM KCl and 0.2% Nonidet P-40) (Volpon et al., 2010) in a total volume of 50 μL at 4 °C for 30 min. Then, the reaction volumes were raised to 500 μl with binding buffer, followed by 20 μl of packed glutathione Sepharose resin (GE Health care, USA) and incubation at 4 °C for 1 h while rotating. Glutathione resin and bound proteins were gently pelleted by centrifugation at 500 ×g for 1 min and washed three times with wash buffer (PBS with 500 mM KCl and 1.0% Nonidet P-40). Proteins were eluted by boiling in 25 μl of SDS loading buffer, and 10 μl was analyzed by 10% denaturing SDS/PAGE and staining with colloidal Coomassie (Sigma, USA).

***In vitro* polymerase activity assay**

An in vitro RNA synthesis assay was performed using a 19-nt template corresponding to the conserved 3' terminus and 20-nt 5' terminus RNA of the viral genome S segment (Supplementary Figure 7). The ds-RNAs were 3' and 5' RNA preannealed at 65 °C before use. 1μg of purified L (final reaction concentration of 0.4 μM) was incubated with 2 μM RNA (3'/5'/ds/3' and 5') in transcription buffer (50 mM Tris-HCl pH 7.0, 40 mM NaCl, 5 mM MnCl2, 10 mM KCl, 1 mM dithiothreitol (DTT), and 0.1 mg/mL BSA) at 25 °C for 30 min. After initial incubation, 1 mM cold ATP/CTP/GTP and 1 μL of [α-32P]-UTP (~0.5 μCi) were added, and the reactions were incubated at 30 °C for 3 h in a final volume of 10 μL. Where indicated, RNA synthesis reactions were supplemented with 2 μM final concentration recombinant Z and Z mutants during L–RNA complex initial incubation. After 3 h incubation, reactions were terminated by 10 μL of 2× stop solution (95% deionized formamide, 20 mM EDTA) and incubation at 95 °C for 2 min. Reactions were analyzed by denaturing gel electrophoresis on a 20% polyacrylamide-urea (7 M) sequencing gel with 0.5× TBE. Gels were exposed for 8 h using a phosphor screen (GE Healthcare, USA), and the radiolabeled RNAs were visualized by a Typhoon FLA 9500 scanner (GE Healthcare, USA).

References

Afonine, P.V., Grosse-Kunstleve, R.W., Echols, N., Headd, J.J., Moriarty, N.W., Mustyakimov, M., Terwilliger, T.C., Urzhumtsev, A., Zwart, P.H., and Adams, P.D. (2012). Towards automated crystallographic structure refinement with phenix.refine. Acta Crystallogr D Biol Crystallogr 68, 352-367.

Emsley, P., Lohkamp, B., Scott, W.G., and Cowtan, K. (2010). Features and development of Coot. Acta Crystallogr D Biol Crystallogr 66, 486-501.

Hastie, K.M., Zandonatti, M., Liu, T., Li, S., Woods, V.L., Jr., and Saphire, E.O. (2016). Crystal Structure of the Oligomeric Form of Lassa Virus Matrix Protein Z. J Virol 90, 4556-4562.

Hu, M., Yu, H., Gu, K., Wang, Z., Ruan, H., Wang, K., Ren, S., Li, B., Gan, L., Xu, S.*, et al.* (2018). A particle-filter framework for robust cryo-EM 3D reconstruction. Nat Methods 15, 1083-1089.

Naydenova, K., and Russo, C.J. (2017). Measuring the effects of particle orientation to improve the efficiency of electron cryomicroscopy. Nat Commun 8, 629.

Peng, R., Xu, X., Jing, J., Wang, M., Peng, Q., Liu, S., Wu, Y., Bao, X., Wang, P., Qi, J.*, et al.* (2020). Structural insight into arenavirus replication machinery. Nature 579, 615-619.

Pettersen, E.F., Goddard, T.D., Huang, C.C., Couch, G.S., Greenblatt, D.M., Meng, E.C., and Ferrin, T.E. (2004). UCSF Chimera--a visualization system for exploratory research and analysis. J Comput Chem 25, 1605-1612.

Rohou, A., and Grigorieff, N. (2015). CTFFIND4: Fast and accurate defocus estimation from electron micrographs. J Struct Biol 192, 216-221.

Scheres, S.H. (2012). RELION: implementation of a Bayesian approach to cryo-EM structure determination. J Struct Biol 180, 519-530.

Tan, Y.Z., Baldwin, P.R., Davis, J.H., Williamson, J.R., Potter, C.S., Carragher, B., and Lyumkis, D. (2017). Addressing preferred specimen orientation in single-particle cryo-EM through tilting. Nat Methods 14, 793-796.

Volpon, L., Osborne, M.J., Capul, A.A., de la Torre, J.C., and Borden, K.L. (2010). Structural characterization of the Z RING-eIF4E complex reveals a distinct mode of control for eIF4E. Proc Natl Acad Sci U S A 107, 5441-5446.

Zheng, S.Q., Palovcak, E., Armache, J.P., Verba, K.A., Cheng, Y., and Agard, D.A. (2017). MotionCor2: anisotropic correction of beam-induced motion for improved cryo-electron microscopy. Nat Methods 14, 331-332.

Supplementary Figures

Supplementary Figure 1

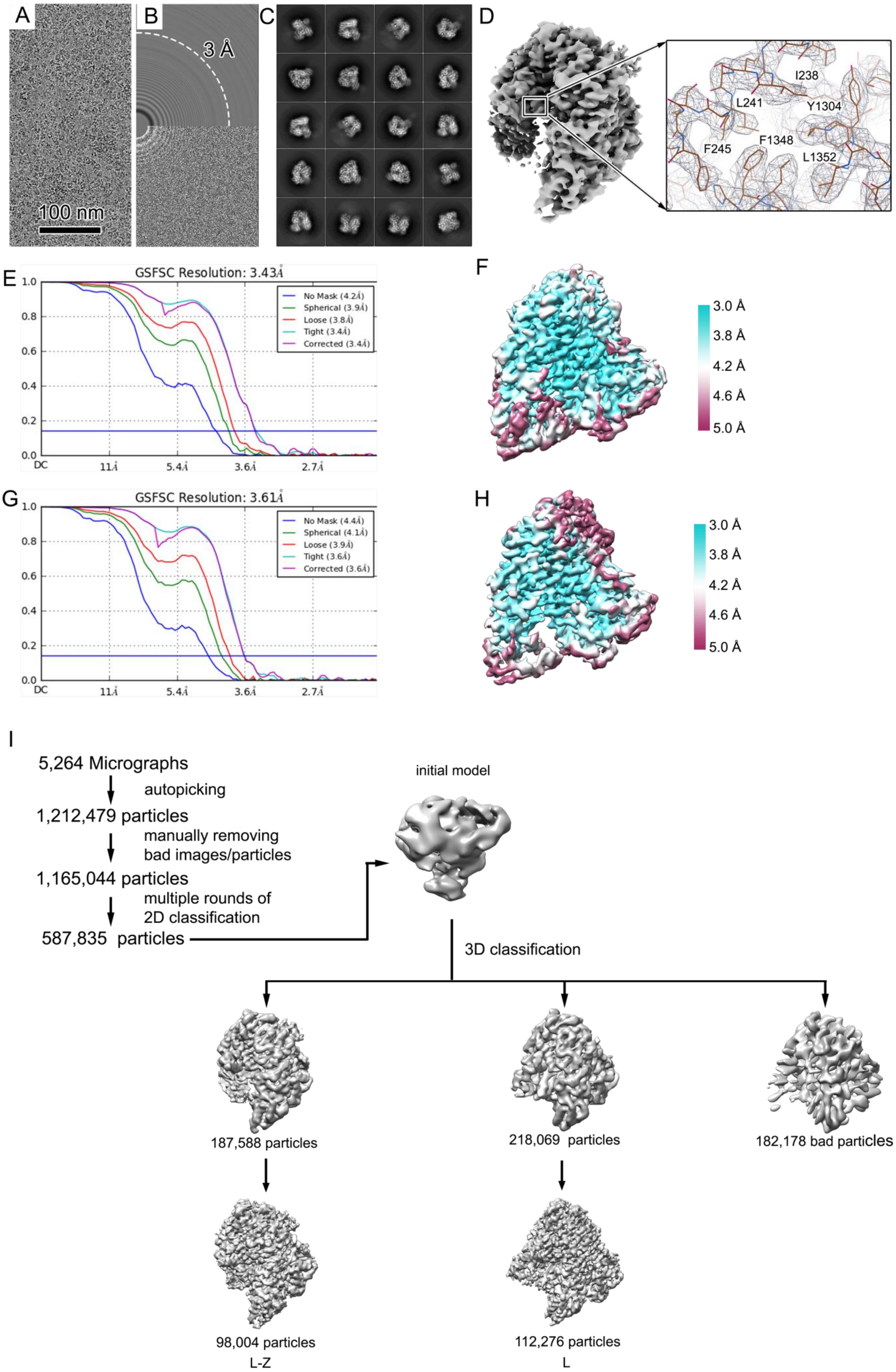
A
B
3 Å
100 nm
C
D
I238
L241
Y1304
F245
F1348
L1352
E
GSFSC Resolution: 3.43Å
No Mask (4.2Å)
Spherical (3.9Å)
Loose (3.8Å)
Tight (3.4Å)
Corrected (3.4Å)
DC
11Å
5.4Å
3.6Å
2.7Å
F
3.0 Å
3.8 Å
4.2 Å
4.6 Å
5.0 Å
G
GSFSC Resolution: 3.61Å
No Mask (4.4Å)
Spherical (4.1Å)
Loose (3.9Å)
Tight (3.6Å)
Corrected (3.6Å)
H
I
5,264 Micrographs
autopicking
1,212,479 particles
manually removing bad images/particles
1,165,044 particles
multiple rounds of 2D classification
587,835 particles
initial model
3D classification
187,588 particles
218,069 particles
182,178 bad particles
98,004 particles
L-Z
112,276 particles
L

Supplementary Figure 1. Cryo-EM reconstruction. (A) Raw image of vitreous ice recorded at defocus values of -1.5 to -2.5 µm. Scale bar, 100 nm. **(B)** Power spectrum of the image shown in (A), with plot of the rotationally averaged intensity versus resolution. The white circle indicates the spatial frequency corresponding to a 3.0 Å resolution. **(C)** Representative class averages. The edge of each square is 238 Å. **(D)** An overview of LCMV L reconstruction is shown in the left panel. A close-up view of a representative region is shown in the right panel. Density is shown as gray mesh; the polypeptide of the refined model is displayed as colored sticks (carbons, gold; nitrogen, blue; and oxygen, red). **(E, G)** Fourier shell correlation (FSC) of the final 3D reconstruction of LCMV L (E) and the L-Z complex (G) following gold standard refinement. FSC curves are plotted before (red) and after (green) masking in addition to post-correction (blue), accounting for the effect of the mask using phase randomization. **(F, H)** Local resolution estimations of LCMV L (F) and the L-Z complex (H) were performed with Relion 2.1 (Scheres, 2012). **(I)** The data processing scheme used to obtain the final map.

Supplementary Figure 2

A

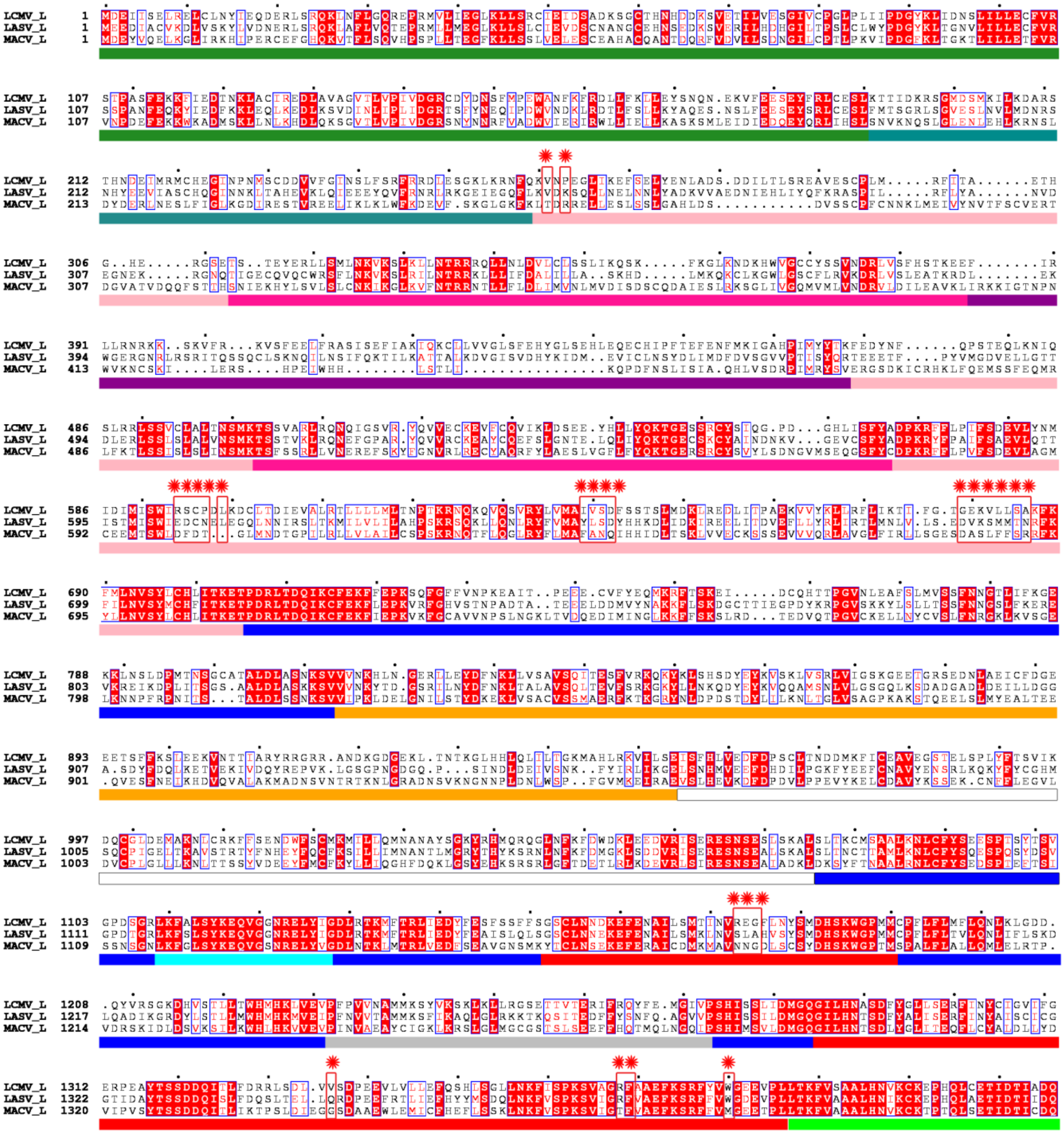

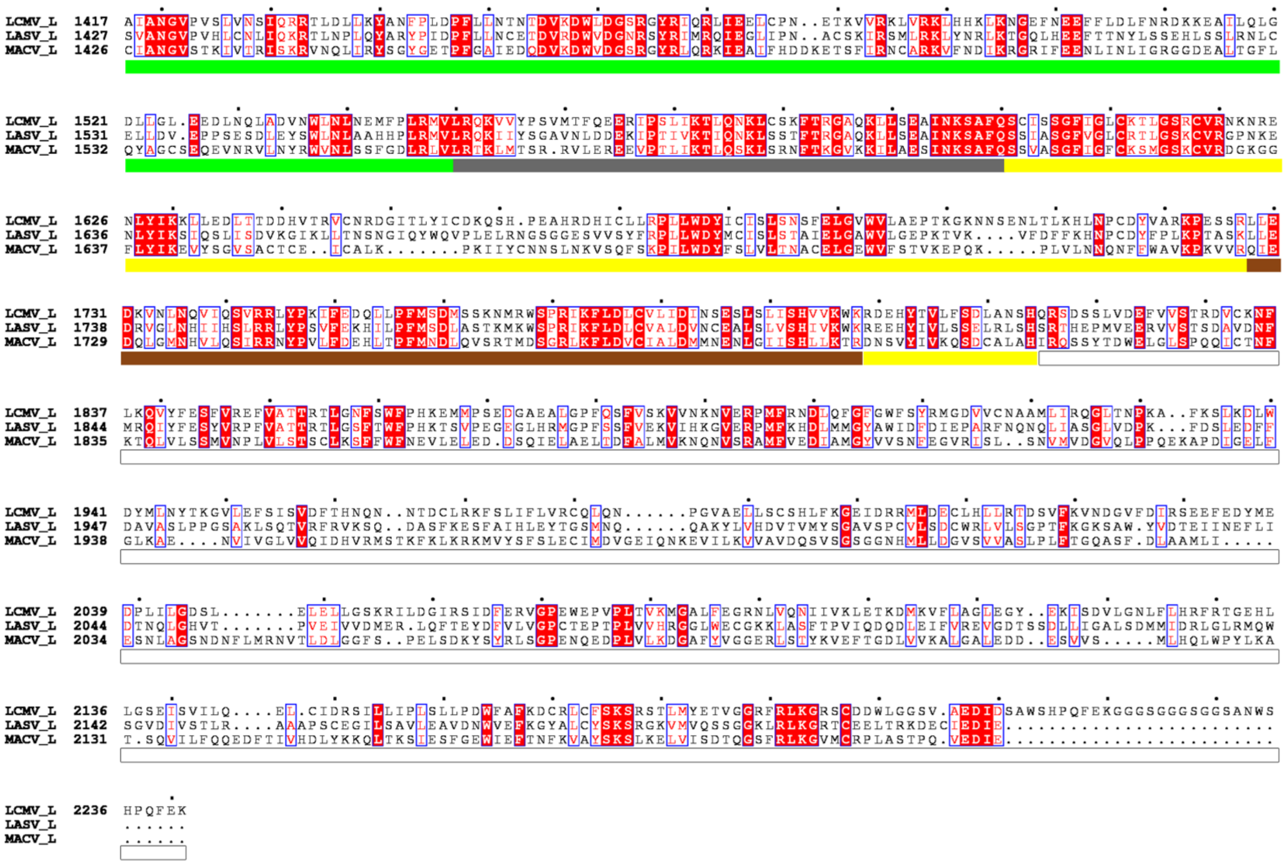

B

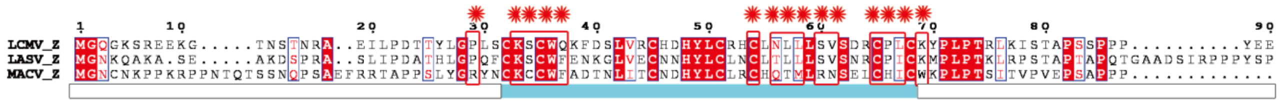

Supplementary Figure 2. Sequence alignment of LCMV, LASV and MACV L and Z proteins. The amino acid sequences of L proteins encoded by LCMV, LASV and MACV are aligned in **(A),** and their Z proteins are aligned in **(B)**. The residues with white labels and red background are the identical residues; the residues with blue frames are the conserved residues; other residues are the less conserved residues. The underlined bars with different colors indicate the domains where the residues are located (in the same color scheme in Figure 1). The residues on the LCMV L-Z interface are highlighted by red frames with red stars.

Supplementary Figure 3

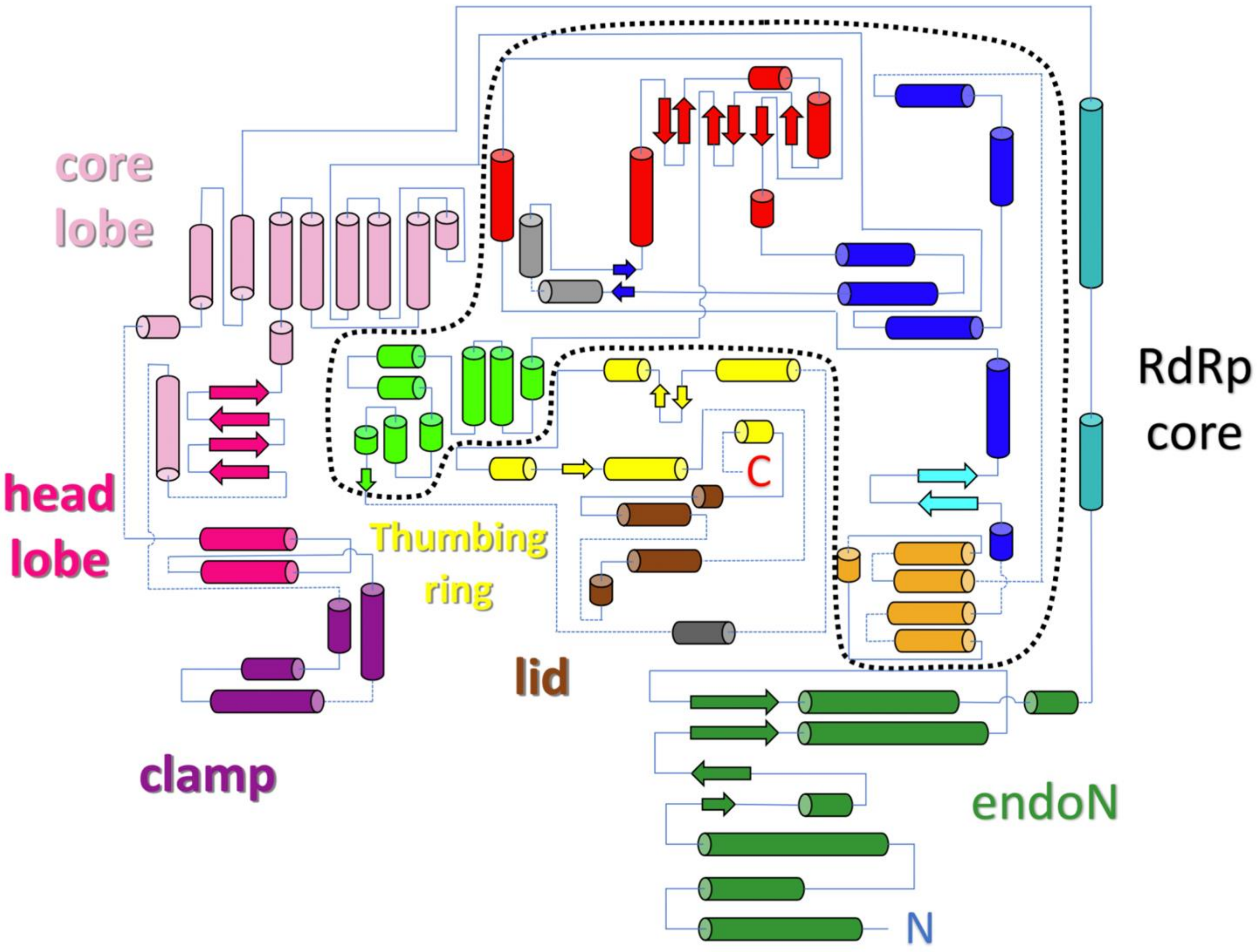

Supplementary Figure 3. Secondary structure diagram of LCMV-L. Secondary structural elements along the LCMV-L sequence are shown as cylinders and arrows for α-helices and β-strands, respectively. Domains are colored as in Figure 1.

Supplementary Figure 4

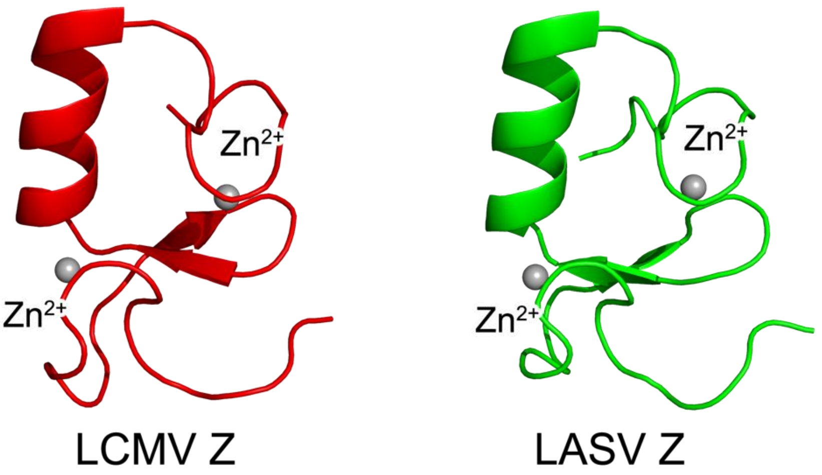

Supplementary Figure 4. Comparison of Z proteins encoded by LCMV and LASV. The structure of LCMV-Z in the LCMV L-Z complex is aligned with the monomeric structure of LASV Z (PDB code: 5I72) (Hastie et al., 2016). Two structures are shown in the same orientation as colored cartoons. The bound zinc ions are represented as gray spheres.

Supplementary Figure 5

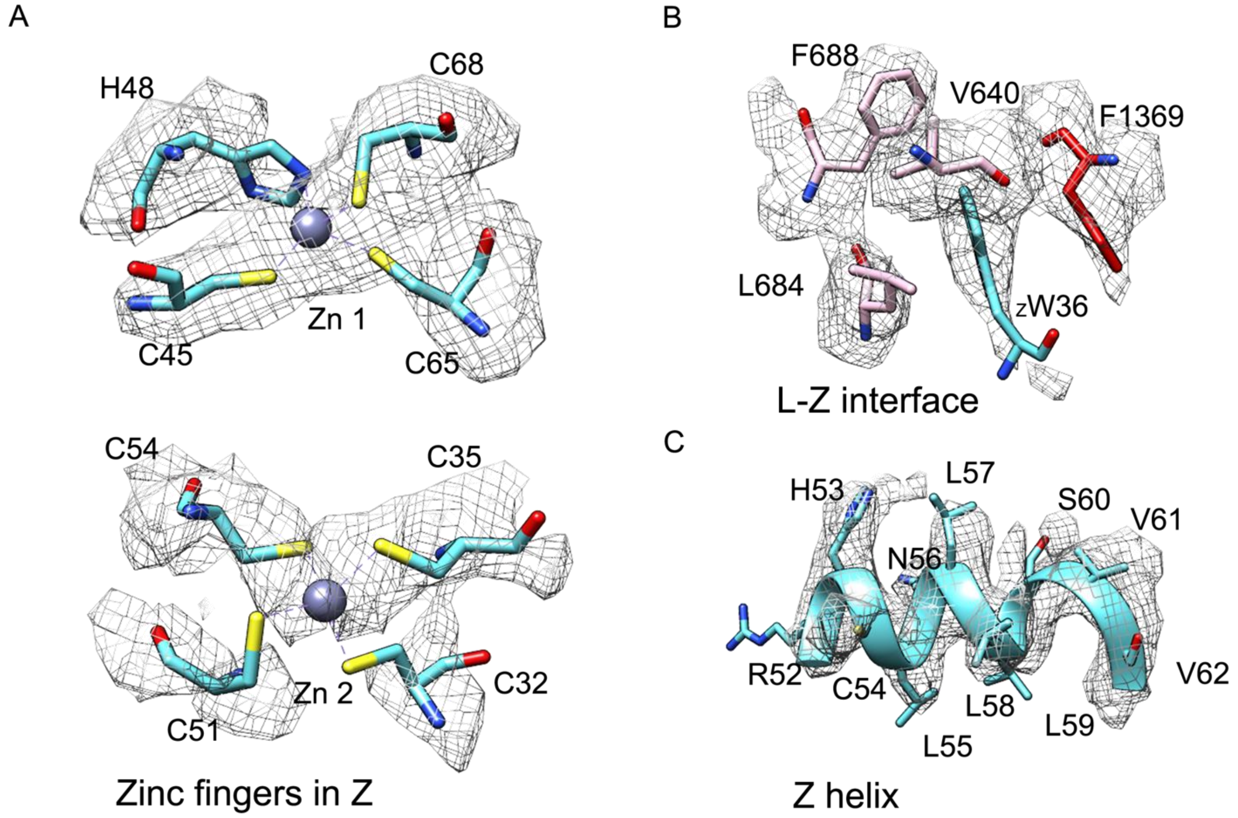

Supplementary Figure 5. Cryo-EM map of Z and Z-L interface. (A, C) The structure and representative density of zinc fingers and helices in Z. The map is shown as a gray mesh, and the bound zinc ions are represented as gray spheres. **(B)** The interface between L and Z. The residues of Z are colored cyan, and the residues of L are colored light pink and red, belonging to the core lobe domain and palm domain.

Supplementary Figure 6

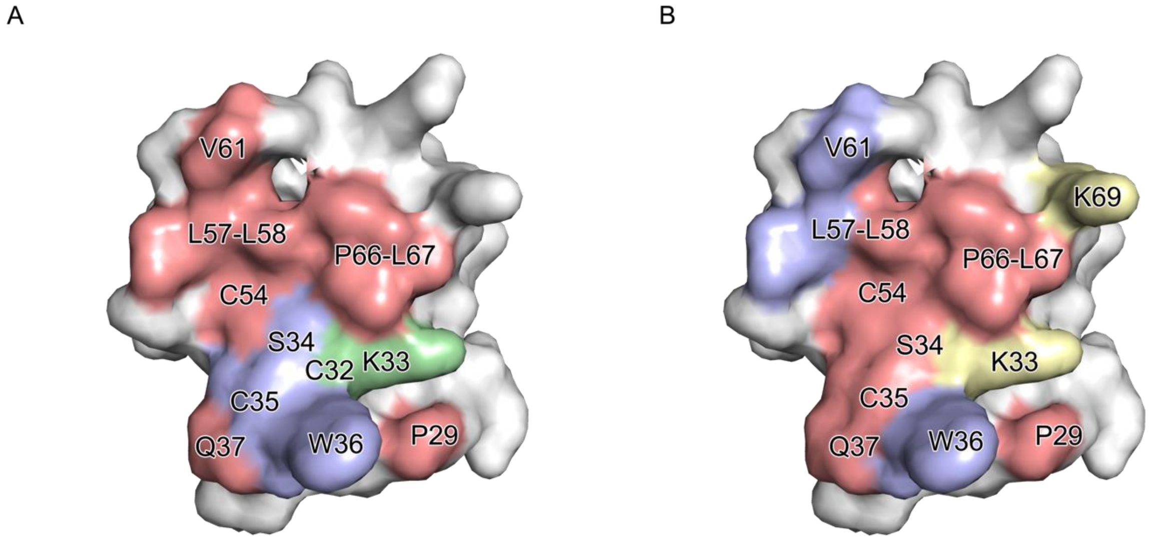

Supplementary Figure 6. Comparison of interaction sites between Z-L, Z-Z and Z-eIF4E. (A) The interaction sites of LCMV Z-L and LCMV Z-eIF4E. **(B)** The interaction sites of LCMV Z-L and LCMV-Z-Z. The interaction sites between LCMV Z-eIF4E and LCMV Z-Z were predicted by homology alignment based on the oligomeric structure of LASV Z and the LASV Z RING-eIF4E complex. The LCMV L-Z interaction sites are colored salmon, the interaction sites of Z-eIF4E are colored pale green, the interaction sites of Z-Z are colored pale yellow, and the light blue region indicates the common interaction sites.

Supplementary Figure 7

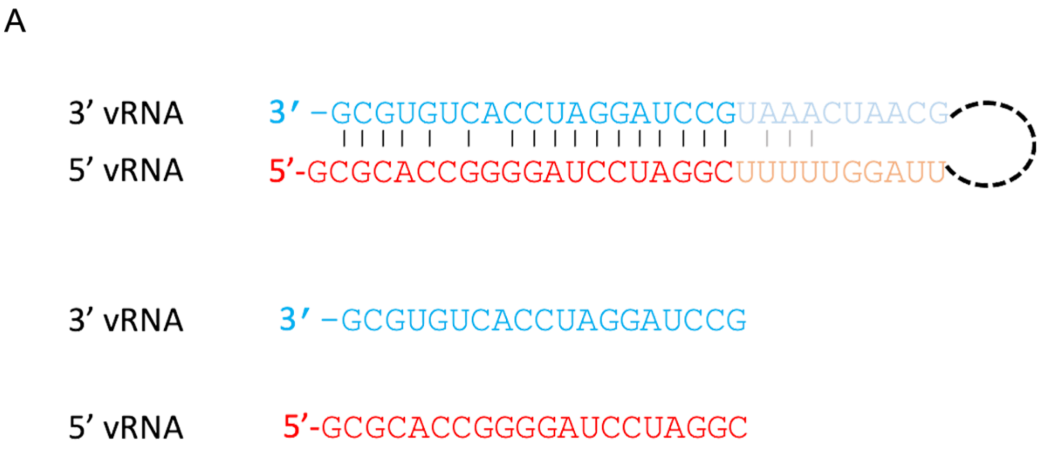

Supplementary Figure 7. vRNA arrangement in the *in vitro* polymerase assay. (A) dsRNA panhandle structure of arenavirus S segment. **(B)** 3’/5’ vRNA sequence used in the polymerase assay.

Supplementary Figure 8

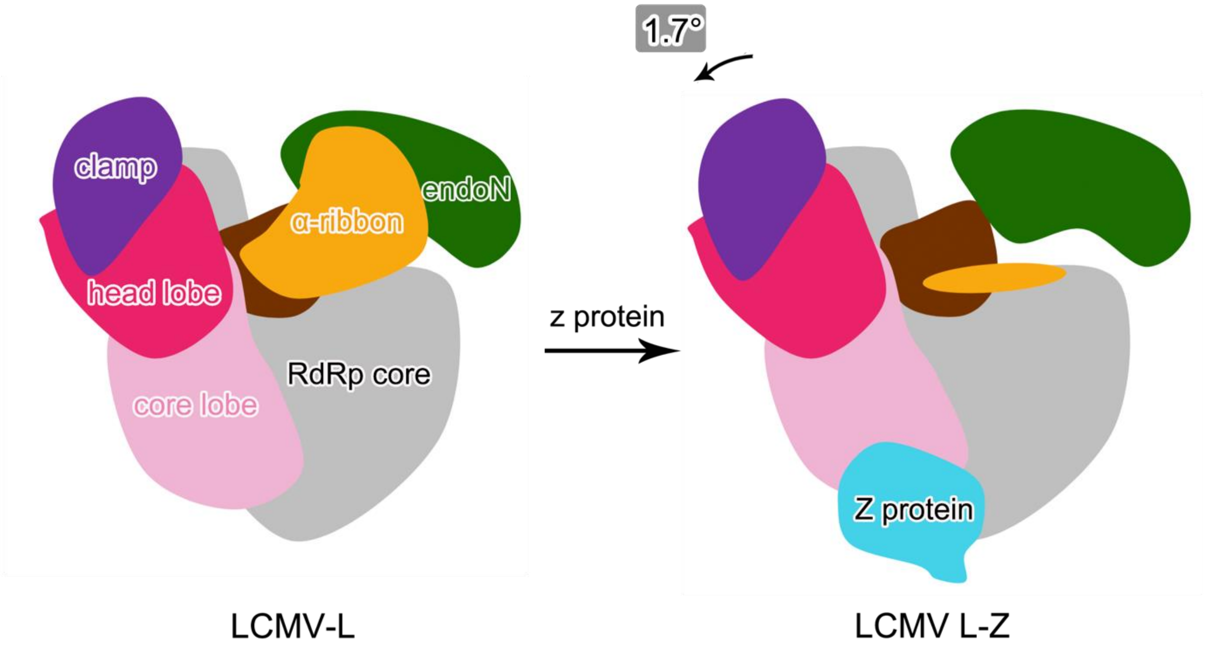

Supplementary Figure 8. Model of LCMV L-Z allosteric regulation. The architecture of LCMV-L displays a compact structure and has an integrated α-ribbon motif**.** After the binding of the Z protein, the core lobe, head lobe, and clamp of the PA-like region have a 1.7° orientation shifting apart from the RdRp core with the binding site of Z as the fixed point, and most parts of the α-ribbon motif lack an interpretable density, suggesting that these parts are in flexible architecture. As described in the text, the binding of Z at the bottom of the interface between the core lobe of the PA-like domain and the palm subdomain of the RdRp domain results in several distinct conformational changes of L structural elements and partially impedes the exit for nascent RNA products; furthermore, it inhibits the RNA synthesis of L.

Supplementary Tables

Supplementary Table 1. Cryo-EM data statistics

	LCMV-L (EMD-33026, PDB 7X6S)	LCMV L-Z (EMD-33028, PDB 7X6V)
**Data collection and processing**		
Magnification	130,000	130,000
Voltage (kV)	300	300
Electron exposure (e–/Å^2)	40.00	40.00
Defocus range (μm)	-2.5 to -1.5	-2.5 to -1.5
Pixel size (Å)	1.08	1.08
Symmetry imposed	C1	C1
Initial particle images (no.)	1,212,479	1,212,479
Final particle images (no.)	112,276	98,004
Map resolution (Å)	3.43	3.61
FSC threshold	0.143	0.143
Map resolution range (Å)	2.3-8.0	3.2-9.0
**Refinement**		
Initial model used (PDB code)	6KLC	6KLC,5I72
Model resolution (Å)	3.43	3.61
FSC threshold	0.143	0.143
Model resolution range (Å)	[illegible] to 3.43	[illegible] to 3.61
Map sharpening *B* factor (Å^2)	-115.3	-112.6
Correlation coefficient between map and model	0.76	0.75
Model composition		
Non-hydrogen atoms	10,988	10926
Protein residues	1453	1430
Ligands	1	3
B factors (Å^2)		
Protein	105.4	58.3
Ligand	20	57.4
R.m.s. deviations		
Bond lengths (Å)	0.003	0.003
Bond angles (°)	0.76	0.66
Validation		
MolProbity score	2.30	2.32
Clashscore	11.22	12.56
Poor rotamers (%)	0.09	0.36
Ramachandran plot		
Favored (%)	92.01	92.21
Allowed (%)	7.99	7.79
Disallowed (%)	0	0

Supplementary Table 2. The L-Z interaction

Z Residues	L Residues
P29	R1173
S34	V682
C35	K681, V682
W36	V640, L684, F688
Q37	K681
C54	V682
L57	C596
L58	C596
V61	C596, L599
P66	L683
L67	L683

Reference

Afonine, P.V., Grosse-Kunstleve, R.W., Echols, N., Headd, J.J., Moriarty, N.W., Mustyakimov, M., Terwilliger, T.C., Urzhumtsev, A., Zwart, P.H., and Adams, P.D. (2012). Towards automated crystallographic structure refinement with phenix.refine. Acta Crystallogr D Biol Crystallogr 68, 352-367.

Emsley, P., Lohkamp, B., Scott, W.G., and Cowtan, K. (2010). Features and development of Coot. Acta Crystallogr D Biol Crystallogr 66, 486-501.

Hastie, K.M., Zandonatti, M., Liu, T., Li, S., Woods, V.L., Jr., and Saphire, E.O. (2016). Crystal Structure of the Oligomeric Form of Lassa Virus Matrix Protein Z. J Virol 90, 4556-4562.

Hu, M., Yu, H., Gu, K., Wang, Z., Ruan, H., Wang, K., Ren, S., Li, B., Gan, L., Xu, S., *et al.* (2018). A particle-filter framework for robust cryo-EM 3D reconstruction. Nat Methods 15, 1083-1089.

Naydenova, K., and Russo, C.J. (2017). Measuring the effects of particle orientation to improve the efficiency of electron cryomicroscopy. Nat Commun 8, 629.

Peng, R., Xu, X., Jing, J., Wang, M., Peng, Q., Liu, S., Wu, Y., Bao, X., Wang, P., Qi, J., *et al.* (2020). Structural insight into arenavirus replication machinery. Nature 579, 615-619.

Pettersen, E.F., Goddard, T.D., Huang, C.C., Couch, G.S., Greenblatt, D.M., Meng, E.C., and Ferrin, T.E. (2004). UCSF Chimera--a visualization system for exploratory research and analysis. J Comput Chem 25, 1605-1612.

Punjani, A., Rubinstein, J.L., Fleet, D.J., and Brubaker, M.A. (2017). cryoSPARC: algorithms for rapid unsupervised cryo-EM structure determination. Nat Methods 14, 290-296.

Rohou, A., and Grigorieff, N. (2015). CTFFIND4: Fast and accurate defocus estimation from electron micrographs. J Struct Biol 192, 216-221.

Scheres, S.H. (2012). RELION: implementation of a Bayesian approach to cryo-EM structure determination. J Struct Biol 180, 519-530.

Tan, Y.Z., Baldwin, P.R., Davis, J.H., Williamson, J.R., Potter, C.S., Carragher, B., and Lyumkis, D. (2017). Addressing preferred specimen orientation in single-particle cryo-EM through tilting. Nat Methods 14, 793-796.

Volpon, L., Osborne, M.J., Capul, A.A., de la Torre, J.C., and Borden, K.L. (2010). Structural characterization of the Z RING-eIF4E complex reveals a distinct mode of control for eIF4E. Proc Natl Acad Sci U S A 107, 5441-5446.

Zheng, S.Q., Palovcak, E., Armache, J.P., Verba, K.A., Cheng, Y., and Agard, D.A. (2017). MotionCor2: anisotropic correction of beam-induced motion for improved cryo-electron microscopy. Nat Methods 14, 331-332.
